# (Re)criminalization of drug use: dynamics and perspectives

**DOI:** 10.1590/0102-311XEN081624

**Published:** 2024-07-22

**Authors:** Francisco I. Bastos

**Affiliations:** 1 Instituto de Comunicação e Informação Científica e Tecnológica em Saúde, Fundação Oswaldo Cruz, Rio de Janeiro, Brasil.

## Clearing up some misunderstandings

Before addressing the (re)criminalization of drug use, we must clarify the distorted (re)emergence of certain points in the legal and scientific fields, including public health.

Firstly, no country has breached international agreements concerning international trafficking and the illicit nature of certain psychoactive substances, called drugs ^1^.

Observed changes in drug policies conserve these Treaties. In national State policies, such as Portugal, possession for personal consumption is not criminalized, but it is subject to psychosocial intervention and, in case of recidivism, to noncriminal sanctions ^2^ ^,^ ^3^.

Changes in drug policies that, in addition to possession for personal consumption, involve the nature of markets refer to national legislation. Changes regarding the rules of use and the market are restricted to *Cannabis* and derivatives. Such is the case of differing policies such as those of US states and policies adopted in Uruguay and the Netherlands. We intend not to discuss their specificities, only to underline that none violates international treaties, none is formally ratified by the respective national States, and should not be understood as a supposed broad drug “liberation” or specifically of cannabis. Although local laws differ from each other, they all include clear rules which can be consulted in articles and reports, such as between member States of the European Community ^4^.

Below we will discuss the paradigmatic cases of the US state of Oregon, the province of British Columbia in Canada, and Brazil.

## Oregon: decriminalization by Referendum followed by (re)criminalization, by legislative decision

Oregon is the only US state where a Referendum (n. 11/2020) decriminalized the possession and use of any psychoactive substance including fentanyl, the opioid that characterizes the 4th wave of opioid spread in the United States ^5^.

What followed was an extremely adverse outcome, combining the biggest crises faced by US society: the housing crisis, acute in California cities but also present in Portland, Oregon’s largest city with 650,000 inhabitants, and a wet and cold climate.

CNN news broadcasted a photo-report on Portland, showing a huge encampment where people living in makeshift tents and in misery, facing adverse weather conditions, used various substances, especially fentanyl. Such negative balance led to the approval of strongly repressive legislation, which not only reverted the situation to that in force before the so-called “measure 110”, but made local legislation stricter than that prior to 2020 ^5^ ^,^ ^6^.

A lesson from such brief and unsuccessful experience is that abrupt changes in drug policy which disregard analyses of the drug scene and the context - urban crisis, the COVID-19 pandemic, and the wide spread of a group of substances (high-potency opioids) - demand from drug policies a capacity that it has never had or will have. In fact, no sectoral policy is capable of abstracting the context in which it is inserted and, in a capitalist country, the market that it intends to modulate.

Quinones’ book ^6^ documents how the US reached the current opioid crisis. Written as a counterpoint piece, chapter by chapter the author alternates the transformations of the illicit heroin market with the successive failures in regulating the pharmaceutical industry, begining with the widespread use of Oxycodone. Both drugs experienced a progressive and intense escalation, and end up colluding in the primarily illicit and the originally pharmaceutical. As an outcome we have the largest supply and consumption of potent opioids ever recorded in history, with more than 100,000 overdoses per year in 2022, according to the Centers for Disease Control and Prevention.

Political decisions, whether the result of direct voting or legislative, that disregard the context and the local (and national) market for any product, especially potentially lethal psychoactive substances, are doomed to failure. Unfavorable contexts and markets react negatively, discrediting well-founded proposals. Oregon’s experience will serve as a banner for a reemerging repression in different contexts.

## British Columbia: public space as a dimension in the dynamics of ongoing (re)criminalization

Canada’s province of British Columbia, especially the city of Vancouver, has the largest number of successful interventions addressing the harmful consequences of substance use. How, then, can we explain the recent partial reversal of current policies ^7^?

As expressed in the Canadian government’s official documentation, changes undertook in Canada are nothing comparable to the sudden changes recorded in Oregon. British Columbia has decades of diverse therapeutic, harm reduction, and shelter and social support actions offered to a significant contingent of substance users, with a strong geographic concentration and in certain social/ethnic strata.

Hundreds of scientific articles document these initiatives, but I draw on psychiatrist Gabor Maté’s book ^8^. Maté combines realism and empathy in describing his experience working with patients with severe addiction. He goes beyond the patients’ conjunctural changes and life contexts, diving deep into their life stories.

British Columbia authorities ruled on the prohibition of drug use in public places, which is commonplace in tobacco control policies in many different societies.

But unlike tobacco use, a habit incorporated into the social imaginary of several countries, the use of certain drugs, especially high-potency opioids and various injectable drugs, is associated with the idea of threat and disorder. Here I turn to Sampson, whose classic work ^9^ uses refined statistics and the exhaustive observation of urban scenes.

One of Sampson’s conclusions is that the perceived disturbance of order has little correlation with the respective metrics. This analysis crowns 30 years of observation and visual recording, followed by a quantitative analysis of what the author calls “ecometrics” (in analogy to the methodological rigor applied to measuring individual variables - psychometrics).

Sampson goes further: although the perceived disturbance of order has a relative empirical basis, it is one of the most powerful drivers of urban ghettos, racism and stigmatization of individuals, communities and places. It is a vicious circle that spans generations. This was captured and analyzed by a series of different studies spanning decades, with several cross-sectional sections and tens of thousands of interviews, both of probabilistic samples of all Chicago (United States) neighborhoods residents and of hundreds of community leaders. Among the other components of what is the most comprehensive urban sociology study, we highlight the systematic observation and recording of thousands of places and events.

Despite sounding like a step backwards, the Canadian authorities’ decisions is in line with the most complete study ever conducted, partially replicated in other cities, with the nuances of a globalized world but which retains a strong local dimension ^9^.

## Brazil: from a complex uncertainty to a possible ongoing criminalization

Brazil’s current legislation regarding the possession and personal use of drugs could be informally described as a legislation of selective decriminalization of drug use and possession.

The main article on this matter (Art. 33 of *Law n. 11,343/2006*) is ambiguously worded, and its enforcement has led to contradictory applications ^10^, to the detriment of people belonging to less favored social strata, black, living in impoverished locations. Such lack of definition led to disparate interpretations and the issue was presented before the Brazilian Federal Supreme Court (STF, acronym in Portuguese), where the judgment on its merits extends for a decade.

Currently, the definition of who is a drug user and therefore, in principle, subject to treatment, and who is a trafficker and, therefore, detained and subject to criminal prosecution, is up to a broad scope of social actors. These range from the police officer on the streets to the judge, from various instances (even the highest court in the country). It was a concrete and paradigmatic case of sentencing, which went through the most diverse instances of Brazilian justice until it reached the STF.

At the time of writing (May, 2024), the current bill approved by the Federal Senate shortens this extensive line since by reinforcing criminalization, it confers discretionary power to security force agents. The bill must still be appreciated by the Chamber of Deputies, but if preserved we can project consequences with historic-structural dimensions.

Regarding the perceived social disorder in places such as downtown São Paulo, the new legislation will support repressive actions which, however, will inevitably retain a strongly local dimension. Comparative studies of US cities at the height of the War on Drugs, during the Nixon era (1969-1974), show heterogeneous implementation of the federal policy of strict compression of the drug supply (after all, it was, metaphorically and operationally, a war).

Werthman and Piliavin’s conclusions, classic authors of the 1960s, unfortunately not available by physical or virtual means on Brazilian websites and libraries, are worth reproducing. I quote here, verbatim, Sampson’s synthesis in which several widely reported episodes of racial discrimination on the part of police officers serves to ratify: “*The police divide up their territories they patrol into readily understandable, and racially tinged, categories. The result is a process of what they* [Werthman & Piliavin] *called ‘ecological contamination’, whereby all persons encountered in a ‘bad’ neighborhood are viewed as possessing the moral liability of the neighborhood itself*” ^9^ (p. 133).

Shortening and reinforcing the line of criminalization has therefore historically resulted in mass incarceration of the poor, people of color, and residents of disadvantaged communities. To a large extent, these characteristics overlap in particular individuals and communities, reinforcing a process already underway.

## Conclusions and perspectives

Prospects for changes in drug policy that favor demand reduction over an emphasis on squeezing supply through criminal policy are more modest today than they were years ago.

Contributing to this is daily disinformation, mixing fact and fiction, confusing different concepts through ignorance or bad faith, as well as a lack of interest in detailed analysis of contexts and substances, contrary to what organizations like Transform have been doing for years, whose numerous publications available for download are ignored by governments, parliamentarians and activists, for and against each of the possible drug policies. One of its publications ^11^ has become a reference for a small group of drug policy students due to the analytical breadth and depth.

It is as if the work of decades of foundations and dozens of universities and research centers were disposable, pressed between radicals on one extreme and the other, incapable not only of dialogue but of paying attention to empirical evidence and analysis based on different disciplines, such as economics, sociology, law, political science, toxicology, psychiatry and neurosciences, etc.

Habermas’ proposal of a world in which one can build a public space through frank and transparent dialog between peers sounds utopian, but one that is a beacon of what is best in us, human beings living in society ^12^. Although I am unaware of any of the philosopher’s texts on drug policy, if he did look into the subject, he must have realized the impossibility of his paradigm, made impossible by radicalisms that disallow a public space from really existing.
