# Supplementary material for: (Re)criminalization of drug use: dynamics and perspectives
Source: Cad Saude Publica. 2024 Jul 22;40(7):e00081624. doi: 10.1590/0102-311XEN081624 (PMC11286261; doi:10.1590/0102-311XEN081624)
Supplement: Translation

# Translation: A ressurgência da criminalização do uso de drogas: dinâmica e perspectivas

**DOI:** 10.1590/0102-311XPT081624

**Authors:** Francisco I. Bastos

**Affiliations:** 1 Instituto de Comunicação e Informação Científica e Tecnológica em Saúde, Fundação Oswaldo Cruz, Rio de Janeiro, Brasil.

## Clarificando alguns mal-entendidos

Antes de abordar a (re)criminalização do uso de drogas, cabe esclarecer pontos que (re)emergem de forma distorcida, mesmo no mundo jurídico e das ciências, incluindo a Saúde Coletiva.

É preciso observar que em nenhum país houve quebra dos acordos internacionais, que dizem respeito ao tráfico e à natureza ilícita de determinadas substâncias psicoativas, denominadas drogas ^1^.

As mudanças observadas nas políticas de drogas preservam esses tratados. Em políticas na órbita de Estados nacionais, como Portugal, o porte para consumo pessoal não é criminalizado, mas é passível de intervenção psicossocial e, em caso de reincidência, de sanções não criminais ^2^ ^,^ ^3^.

As alterações nas políticas de drogas que, além do porte para consumo pessoal, envolvem a natureza dos mercados, referem-se a legislações infranacionais. As mudanças referentes às normas de uso e ao mercado se restringem à *Cannabis* e derivados. É o caso de políticas díspares entre si, como a de estados norte-americanos e políticas adotadas no Uruguai e nos Países Baixos. Não cabe discutir suas especificidades, mas sublinhar que nenhuma viola tratados internacionais, nenhuma é formalmente ratificada pelos respectivos Estados nacionais e não devem ser compreendidas como uma suposta “liberação” das drogas, em sentido amplo ou especificamente da *Cannabis*. Embora as legislações locais difiram entre si, todas contemplam regras claras, que podem ser consultadas em artigos e relatórios, como entre Estados membros da Comunidade Europeia ^4^.

Trataremos, neste artigo, sobre casos paradigmáticos referentes ao estado norte-americano do Oregon, à Província da Colúmbia Britânica, no Canadá, e ao Brasil.

## Oregon: descriminalização, por referendo, seguida de (re)criminalização, por decisão legislativa

O Oregon é o único estado norte-americano em que um referendo (nº 11/2020) descriminalizou a posse e uso de qualquer substância psicoativa, inclusive do opioide que caracteriza a quarta onda da disseminação de opioides nos Estados Unidos, o fentanil ^5^.

O desfecho foi extremamente adverso, conjugando as maiores crises enfrentadas pela sociedade norte-americana: a crise de moradia, aguda em cidades da Califórnia, mas também presente em Portland, maior cidade de Oregon, com 650 mil habitantes; e um clima úmido e frio.

A rede de notícias CNN disponibilizou fotorreportagem sobre Portland, que mostrava um imenso acampamento, no qual pessoas vivendo em situação de miséria e enfrentando condições climáticas adversas utilizavam diversas substâncias, especialmente o fentanil. O saldo negativo acabou por motivar a aprovação de legislação fortemente repressiva, que não apenas reverteu a situação àquela vigente antes da chamada “medida 110”, mas tornou a legislação local mais rígida que a anterior a 2020 ^5^ ^,^ ^6^.

Uma lição dessa experiência tão breve e malsucedida é que mudanças bruscas na política de drogas, que não levam em consideração as análises da cena de drogas e do contexto - crise urbana, a pandemia da COVID-19 e a ampla disseminação de um grupo de substâncias (os opioides de alta potência) - demandam das políticas de drogas uma capacidade que ela jamais teve ou terá. Aliás, nenhuma política setorial é capaz de abstrair o contexto em que está inserida e, em um país capitalista, o mercado que tentará modular.

O livro de Quinones ^6^ documenta como os Estados Unidos chegaram à atual crise de opioides. Escrito ao modo de uma composição musical que se vale do contraponto, o autor alterna, capítulo a capítulo, as transformações do mercado ilícito de heroína e as sucessivas falhas na regulação da indústria farmacêutica, que se inicia com o uso disseminado da oxicodona. Ambas experimentam uma progressiva e intensa escalada e acabam por confluir na combinação dos dois mercados, o primariamente ilícito e o originalmente farmacêutico. O desfecho é a maior oferta e consumo de opioides potentes já registrados na história, com mais de 100 mil óbitos por overdose no ano, em 2022, segundo o Centro de Controle e Prevenção de Doenças dos Estados Unidos.

Decisões políticas, sejam elas fruto do voto direto ou legislativas, que não observam em detalhe o contexto e o mercado local (e nacional) de qualquer produto, em especial substâncias psicoativas potencialmente letais, estão destinadas ao fracasso. Contextos e mercados desfavoráveis retroagem de forma negativa, desacreditando proposições bem-fundamentadas. A experiência de Oregon servirá de bandeira a um recrudescimento da repressão em contextos completamente distintos.

## Colúmbia Britânica: a dimensão do espaço público na dinâmica da (re)criminalização em curso

A província canadense de Colúmbia Britânica, em especial a cidade de Vancouver, contempla a rede urbana que conta com o maior número de intervenções bem-sucedidas de enfrentamento das consequências danosas do uso de substâncias. Como explicar, então, a recente reversão parcial das políticas em curso ^7^?

Conforme expresso na documentação oficial do governo canadense, não se trata de nada comparável às mudanças bruscas registradas em Oregon. A Colúmbia Britânica conta com décadas de diversas ações terapêuticas, de redução de danos e de acolhimento e suporte social, oferecidas a um contingente expressivo de usuários de diversas substâncias, com forte concentração geográfica e em determinados estratos sociais/étnicos.

Centenas de artigos científicos documentam essas iniciativas, mas recorro ao livro do psiquiatra Gabor Maté ^8^, que combina realismo e empatia na descrição de sua experiência com a clínica de pacientes com quadros graves de dependência. Maté vai além das mudanças conjunturais dos pacientes e seus contextos de vida, mergulhando fundo nas suas histórias de vida.

A decisão das autoridades da Colúmbia Britânica se refere à proibição do uso de drogas em locais públicos, o que é lugar comum nas políticas de controle do tabagismo nas mais diferentes sociedades.

Mas, diferentemente do uso do tabaco, hábito incorporado ao imaginário social de diversos países, o uso de determinadas drogas, especialmente opioides de alta potência e drogas injetáveis, está associado à ideia de ameaça e desordem. Recorro aqui a Sampson, cuja obra clássica ^9^ lança mão de estatística refinada, assim como da observação exaustiva das cenas urbanas. Uma de suas conclusões é que a perturbação da ordem percebida tem pouca correlação com as métricas a ela referentes. Essa análise coroa 30 anos de trabalho de observação e registro visual, seguido de uma análise quantitativa do que o autor denomina “ecometria” (em analogia ao rigor metodológico aplicado à mensuração de variáveis individuais [psicometria]).

Sampson vai mais longe: apesar de a perturbação da ordem percebida ter base empírica relativa, constitui um dos mais poderosos motores da formação de guetos urbanos, do racismo e da estigmatização de indivíduos, comunidades e locais. Há um círculo vicioso que atravessa gerações. Isso foi capturado e analisado no âmbito de um conjunto de diferentes estudos que se estenderam por décadas, com diversos cortes seccionais e dezenas de milhares de entrevistas, tanto de amostras probabilísticas de moradores de todos os bairros de Chicago (Estados Unidos) como de centenas de lideranças. Entre os outros componentes do mais abrangente estudo de sociologia urbana, cabe destacar a observação e o registro sistemático de milhares de locais e eventos.

Embora soe como um recuo, a decisão das autoridades canadenses está em sintonia com o mais completo estudo já realizado, replicado parcialmente em outras cidades com as nuances próprias de um mundo globalizado, mas que conserva uma forte dimensão local ^9^.

## Brasil: de uma complexa indefinição a uma possível criminalização em curso

A atual legislação brasileira referente ao porte e uso pessoal de drogas poderia ser descrita de uma maneira não formal como uma legislação de descriminalização seletiva do porte e uso pessoal de drogas.

O artigo central referente a essa matéria (art. 33 da *Lei nº 11.343*, de 23 de agosto de 2006) é de redação ambígua, o que tem causado aplicações contraditórias ^10^, em detrimento de pessoas pertencentes a estratos sociais menos favorecidos, residentes em localidades pobres e negras. Essa indefinição levou a interpretações díspares, e a questão foi levada ao Supremo Tribunal Federal (STF), em que o julgamento do seu mérito se estende por uma década.

Por ora, a definição de quem seria usuário de drogas e, portanto, objeto de tratamento, e quem seria traficante, portanto, detido e objeto de processo criminal, cabe a uma extensa linha de atores sociais. Esses vão desde o policial que atua nas ruas até o juiz, de diversas instâncias (mesmo da mais alta corte do país). Foi um caso concreto de penalização, que se tornou paradigmático e percorreu as mais diversas instâncias da justiça brasileira até chegar ao STF.

O atual projeto, até o momento de redação deste texto (maio de 2024), aprovado pelo Senado Federal encurta essa extensa linha, pois, ao reforçar a criminalização, confere um poder discricionário aos agentes das forças de segurança. O projeto passará pela Câmara dos Deputados, mas, caso preservado, é possível prever consequências com dimensões histórico-estruturais.

No que tange à percepção de desordem social em locais como a região central de São Paulo, a nova legislação dará respaldo às ações repressivas, que conservarão, inevitavelmente, uma dimensão fortemente local. Os estudos comparativos de cidades norte-americanas, no auge da Guerra às Drogas na era Nixon (1969-1974), mostram a heterogeneidade na aplicação de uma política federal de estrita compressão da oferta de drogas (afinal, tratava-se, metafórica e operacionalmente, de uma guerra).

Cabe reproduzir as conclusões de Werthman e Piliavin, autores clássicos dos anos 1960, infelizmente indisponíveis por meios físicos ou virtuais em *sites* e bibliotecas brasileiras. Cito aqui, literalmente, a síntese de Sampson ^9^ (p. 133), em que diversos episódios de discriminação racial por parte de policiais, amplamente noticiado, só fez ratificar: “*A polícia divide os territórios por ela patrulhados entre aqueles de fácil discernimento e aqueles de marcada estratificação racial. Esta divisão define categorias distintas. O resultado é um processo que* [Werthman e Piliavin] *denominam ‘contágio ecológico’, por meio do qual toda e qualquer pessoa identificada em um território definido como ‘ruim’ é vista como possuidora da carga moral atribuída ao respectivo território*” (tradução livre).

Portanto, encurtar e reforçar a linha da criminalização resulta, historicamente, em encarceramento em massa de pobres, negros e residentes de comunidades desfavorecidas. Em larga medida, essas características se sobrepõem a determinados indivíduos e comunidades, reforçando um processo já em curso.

## Conclusões e perspectivas

As perspectivas a respeito das mudanças nas políticas de drogas que privilegiam a redução da demanda, em detrimento da ênfase em comprimir a oferta via política criminal, são hoje mais modestas do que as de anos atrás.

Contribuem para isso a desinformação cotidiana, que mistura fato e ficção, embaralhando, por ignorância ou má-fé, conceitos distintos, além de um desinteresse por análises minuciosas de contextos e substâncias, na contramão do que organizações vêm fazendo há anos, como a Transform, cujas inúmeras publicações, disponíveis para *download*, são ignoradas por governos, parlamentares e ativistas, pró e contra cada uma das possíveis políticas de drogas. Uma das suas publicações ^11^ se tornou referência para um pequeno grupo dos que estudam as políticas de drogas, pela amplitude e profundidade das análises.

É como se um trabalho de décadas de fundações e de dezenas de universidades e centros de pesquisa fossem descartáveis, prensados entre radicais de um extremo e de outro, incapazes não apenas de dialogar, como também de prestar atenção às evidências empíricas e às análises fundamentadas nas diferentes disciplinas, como economia, sociologia, direito, ciência política, toxicologia, psiquiatria e as neurociências etc.

A proposta de Habermas de um mundo em que seja possível construir o espaço público por meio do diálogo franco e transparente entre pares é uma perspectiva utópica, mas balizadora do que pode haver de melhor em nós, seres humanos, vivendo em sociedade ^12^. Embora não conheça nenhum texto do filósofo sobre a política de drogas, suponho que, se ele se debruçou sobre o tema, deve ter constatado a impossibilidade de seu paradigma, inviabilizado pelos radicalismos que sequer permitem que um espaço público exista de fato.
